# Role of MEK partner-1 in cancer stemness through MEK/ERK pathway in cancerous neural stem cells, expressing EGFRviii

**DOI:** 10.1186/s12943-017-0703-y

**Published:** 2017-08-22

**Authors:** Soo-Jung Kwon, Ok-Seon Kwon, Keun-Tae Kim, Young-Hyun Go, Si-in Yu, Byeong-ha Lee, Hiroyuki Miyoshi, Eunsel Oh, Seung-Ju Cho, Hyuk-Jin Cha

**Affiliations:** 1 College of Natural Sciences, Department of Life Sciences, Sogang University, Seoul, 121-742 South Korea; 2 Subteam for manipulation of cell fate, RIKEN BioResource Center, Wako, Japan; 3 Laboratory of Cancer Genomics and Molecular Pathology, Samsung Biomedical Research Institute, Samsung Medical Center, Seoul, South Korea

**Keywords:** Glioblastoma multiforme (GBM), *LAMTOR3*, MP1, ERK1/2, Cancer stemness, EGFRviii

## Abstract

**Background:**

Glioma stem cells (GSCs) are a major cause of the frequent relapse observed in glioma, due to their high drug resistance and their differentiation potential. Therefore, understanding the molecular mechanisms governing the ‘cancer stemness’ of GSCs will be particularly important for improving the prognosis of glioma patients.

**Methods:**

We previously established cancerous neural stem cells (CNSCs) from immortalized human neural stem cells (F3 cells), using the H-Ras oncogene. In this study, we utilized the EGFRviii mutation, which frequently occurs in brain cancers, to establish another CNSC line (F3.EGFRviii), and characterized its stemness under spheroid culture.

**Results:**

The F3.EGFRviii cell line was highly tumorigenic in vitro and showed high ERK1/2 activity as well as expression of a variety of genes associated with cancer stemness, such as *SOX2* and *NANOG*, under spheroid culture conditions. Through meta-analysis, PCR super-array, and subsequent biochemical assays, the induction of MEK partner-1 (MP1, encoded by the *LAMTOR3* gene) was shown to play an important role in maintaining ERK1/2 activity during the acquisition of cancer stemness under spheroid culture conditions. High expression of this gene was also closely associated with poor prognosis in brain cancer.

**Conclusion:**

These data suggest that MP1 contributes to cancer stemness in EGFRviii-expressing glioma cells by driving ERK activity.

**Electronic supplementary material:**

The online version of this article (doi:10.1186/s12943-017-0703-y) contains supplementary material, which is available to authorized users.

## Background

A subset of cell populations showing specific biological properties associated with high tumorigenicity and cellular plasticity has been designated cancer stem cells (CSCs) or tumor-initiating cells (TICs). There is emerging evidence that CSCs in glioma [glioma stem cells (GSCs), or glioma stem-like cells (GSLCs)] may be the main cause of poor clinical outcomes due to their resistance to chemo- and radiotherapy [1–3]. Therefore, GSCs are considered an important target for glioma therapy [4], and studies to understand the biology of GSCs are being actively undertaken [5, 6].

Recently, it has been demonstrated that neural stem cells (NSCs) undergoing neoplastic transformation are responsible for glioma formation [7–9]. NSCs are more susceptible to oncogenic transformation than differentiated glial cells [7, 10]. For example, immortalized human fetal NSCs established by the introduction of v-myc (HB.F3 cells) [11], which are widely used as a model system to examine the possible therapeutic effects of genetically modified NSCs or glial cells [12–14], undergo oncogenic transformation by H-Ras (forming cancerous neural stem cells (CNSCS): F3.Ras cells) [10] but not Akt [15]. In particular, oligodendrocytes derived from F3 cells are resistant to oncogenic transformation by H-Ras [10], implying that NSCs are more susceptible to oncogenic transformation than glial cells (e.g., astrocytes and oligodendrocytes). Importantly, F3.Ras cells have similar molecular properties as GSCs [6], implying that common mechanisms control neural cancer stemness in both GSCs and CNSCs.

Amplification and gain-of-function mutations of epidermal growth factor receptor (*EGFR*) are the most common genetic alteration in the brain cancers, with a frequency of approximately 40% in glioma [16]. In particular, the type III EGFR mutation (also called *EGFRviii*, or del2–7 EGFR) results in an in-frame deletion of 267 amino acids from the extracellular domain of EGFR, resulting in constitutive activation [17]. This is the most frequent genetic mutation in glioblastoma multiforme (GBM), with an overall prevalence of 20–30%. Although *RAS*, a downstream effector of EGFR, is one of the most frequently mutated oncogenes in many types of cancer [18], mutations of *RAS* in glioma are relatively rare [19]. Instead of *RAS* mutations, loss-of-function mutations are observed in neurofibromin 1 (*NF1*), a negative regulator of Ras signaling toward MEK/ERK1/2 [19]. Consistent with this model, mice with mutations in *Nf1* and *Trp53* develop astrocytoma [20] with stem cell characteristics [21]. Similarly, dual knockout of phosphatase and tensin homolog (*Pten*) and *Trp53* results in a high-grade malignant glioma that resembles primary human GBM and shows increased NSC self-renewal capacity [22]. Notably, Akt activation due to *PTEN* loss of function [23] and MEK/ERK1/2 activation are both important for the self-renewal and tumorigenicity of GSCs [24]. However, the differences between Akt and MEK/ERK1/2 downstream of EGFR activation have remained less clear in glioma and GSCs.

Late endosomal/lysosomal adaptor, MAPK and MTOR activator 3 (*LAMTOR3*), which encodes MEK partner-1 (MP1), was initially identified as a scaffolding protein for MEK1 and ERK1 that enables ERK1 activation [25]. It has been well characterized as a key regulator of endosomal signaling [26], and a role for MP1 in cancer, via MEK and ERK hyperactivation, has recently been demonstrated in pancreatic tumorigenesis [27].

In this study, another type of CNSCs was established by expression of EGFRviii in F3 NSCs (F3.EGFRviii cells). Although EGFRviii signaled through Akt under adherent culture conditions, EGFRviii signaling through MEK/ERK1/2 became predominant when the cells were cultured under spheroid-inducing conditions, which promote neural cancer stemness. In addition, MP1 was shown to mediate this switch in signaling from Akt to ERK1/2, and therefore promoted the phenotype of neural cancer stemness in F3.EGFRviii cells. Finally, MP1 expression was strongly associated with poor survival in human glioma patients.

## Methods

### Cell culture

F3 cells were maintained as previously described [6, 10]. F3.EGFRviii cells were derived from F3 cells and U87 and 293 T cells were purchased from Korean cell line bank (cellbank.snu.ac.kr/). They were maintained in Dulbecco’s modified Eagle’s medium (DMEM), supplemented with 10% fetal bovine serum (FBS), gentamicin (50 μg/ml), were purchased from Gigbco-BRL at 37 **°**C in a humidified atmosphere of 5% CO_2_ in air.

### Reagents and antibodies

Primary antibodies against EGFR (cat# sc-03), SIRT1 (cat# sc-15,404), β-actin (cat# sc-47,778), α-tubulin (cat# sc-8035) and ERK2 (cat# sc-154) were obtained from Santa Cruz Biotechnology. Primary antibodies against phosphor-EGF Receptor (pEGFR, Tyr1068: cat# 3777X), *LAMTOR3* **(**cat# 8168), phosphorylated AKT (pAKT, ser473: cat# 4060X), phospho**-**p44/42 (pERK, Thr202/Tyr204: cat# 9106), phospho**-**c-Jun (pc-Jun, ser63: cat# 9261), phospho**-**MEK1/2 (pMEK, ser217/221: cat# 9121), Sox2 (cat# 14962X), phospho-p38 MAPK (p38, Thr180/Tyr182: cat# 4631), phosphorylated p90RSK (pRSK, ser380: cat# 9341) and phospho-SAPK/JNK (pJNK, Thr183/Tyr185: cat#9251) were acquired from Cell Signaling Technology. Phorbol-12-myristate 13-acetate (PMA, cat# P1585) and doxycycline hyclate (DOX, cat# D9891) were obtained from Sigma. 0.25% trypsin ethylenediaminetetraacetic acid (EDTA) was purchased from Gigbco-BRL.

### Generation of EGFRviii inducible cell line

CSIV-TRE-RfA-EF-KT lentiviral plasmids expressing doxycycline inducible EGFRviii was constructed using Gateway Technology with Clonase II (Invitrogen) as described previously [28]. The following primers were used: attB-EGFRviii-F: 5′-GGGGACAAGTTTGTACAAAAAAGCAGGCTTCATGCGACCC TCCGGGACGGC-3′, attB-EGFRviii–R: 5′-GGGGACCACTTTGTACAAGAAAG.

CTGGGTTTCATGCTCCAATAAATTCA-3′. The lentiviruses containing doxycycline-inducible EGFRviii was produced using ViraPower Lentiviral expression system in 293FT cells (Invitrogen). Briefly, 293FT cells were transfected with this lentiviral plasmid and two packaging plasmids (pMDL-gpRRE and pCMV-VSVG-RSV-Rev) at a ratio of 2:2:1. The supernatants were collected at 48 h post-transfection, filtered, and then transduced into F3 at a 1:1 ratio twice. FACS sorted the Kusabira-Orange positive cells.

### Quantitative real-time PCR

Total RNA was extracted from cells using Total RNA Extraction Kit (Intron, cat# 17061), and then converted to cDNA using PrimeScript RT Master Mix (Takara, cat# RR036) in accordance with the manufacturer’s instruction. The synthesized cDNAs were used as templates to perform the real-time PCR with Light Cycler 480 Instrument II (Roche) using SYBR Premix Ex Taq (Takara, cat# RR420) under the following conditions: denaturation at 95 °C for 30 s, followed by 40 cycles of 95 °C for 5 s, 58 °C for 15 s, and 72 °C for 20 s. The average threshold cycle for each gene was determined from triplicate reactions and then levels of gene expression relative to TATA-binding protein (TBP) or Ribosomal protein L13a (RPL13A) were determined. Primer pairs were listed on the following table (Additional file 1: Table S1). ERK dependent gene expression was demonstrated by RT^2^ profiler PCR array human MAP kinase Signaling Pathway as described by the manufacturer. The reactions were carried out in an Applied Biosystems 7900HT Fast-Real Time PCR System.

### Sphere culture

F3 and F3.EGFRviii were maintained under neurospheres culture condition that DMEM/F12 supplemented with 2% B27, 8 mM HEPES, 0.1% gentamicin, 20 ng/mL basic fibroblast growth factor (bFGF), and 20 ng/mL epidermal growth factor (EGF) (Invitrogen) for 14 days as described previously [6].

### Lentiviral genes transfer

The shRNA *LAMTOR3* plasmid (SHCLNG-NM_021970) was purchased from Sigma Aldrich. All procedures were according to the manufacturer’s instructions (ViraPower Lentiviral ExpressionSystems, Invitrogen). Each viral plasmid (10 μg: gene of interest, VSVG, 5 μg: Gag/Pol) was transfected into 293Tcells using lipofectamine 2000 (Invitorgen, #11668–027). After 48 h, cultured media containing the viruses were gathered from the transfected 293 T cells and were filtered (0.45 μm filter, Millipore). F3.EGFRviii was incubated with the virus containing media for 24 h with 4 μg/ml of polybrene (Sigma Aldrich). Infected cells were selected by 1 μg/ml of puromycin.

### TCGA analysis

The DNA copy number, mRNA expression and clinical data obtained from about 500 GBM patients were downloaded from the TCGA data portal (https://tcga-data.nci.nih.gov/). Gene expression data were generated by the Agilent microarray chips, and multiple probes were averaged to get a single expression value per gene. DNA copy number data were generated by the Affymetrix SNP6.0 chips, and the segmented copy numbers were averaged by gene. The samples with EGFR amplification were defined by both upregulated mRNA expression levels (≥2folds) and high DNA copy numbers (≥ 8) of EGFR gene. To compare mRNA expression levels of *LAMTOR3* between EGFR-amplified and EGFR-normal samples, t-test was used. Kaplan-Meier survival analysis and log-rank test were performed to estimate and compare survivals of glioblastoma patients by *LAMTOR3* mRNA expression levels. The glioblastoma patients were grouped by the *LAMTOR3* expression levels, which were divided into 3 equal intervals; high, med and low.

### Statistical analysis

Graphical data were presented as mean ± S.D. Statistical significance among three groups and between groups were determined using one- or two-way analysis of variance (ANOVA) following Bonferroni multiple comparisons pos*t*-test and Student’s *t*-test, respectively. Significance was assumed for *P* < 0.05(*), *P* < 0.01(**), and *P* < 0.001(***).

## Results

### Establishment of EGFRviii-expressing human neural stem cells

**Fig. 1:**
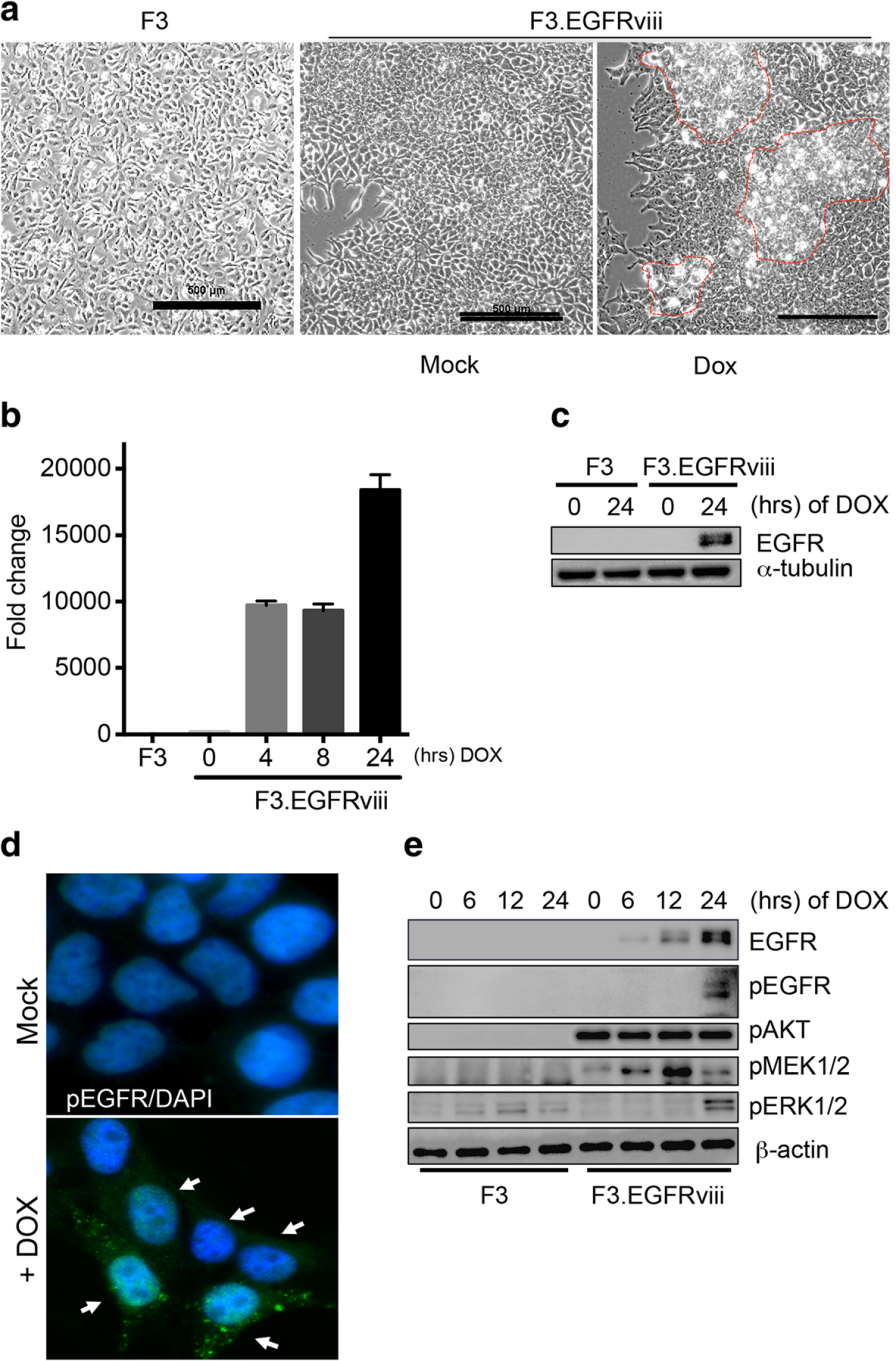
Establishment of EGFRviii-expressing human neural stem cells. **a** Phase-contrast images showing morphological characteristics of parental F3 cells and F3.EGFRviii cells after treatment with Doxycycline (DOX; 1 μg/ml) compared with mock (scale bar = 500 μm). **b** The mRNA expression of EGFRviii in F3 and F3.EGFRviii cells was determined by real-time PCR (*n* = 2). F3.EGFRviii cells were treated with DOX (1 μg/ml) at the indicated times. **c** F3 and F3.EGFRviii cells were treated with DOX (1 μg/ml) and harvested at the indicated times. The level of EGFR was determined by immunoblotting, using α-tubulin as a loading control. **d** F3.EGFRviii cells were immunostained with an antibody to pEGFR (green) after treatment with Dox (1 μg/ml). DAPI (4′,6′-diamidino-2-phenylindole; blue) was used for a nuclear counterstain. The *white arrows* indicate pEGFR-positive cells. **e** F3 and Dox-treated F3.EGFRviii cells (1 μg/ml) were harvested at the indicated times and subjected to immunoblot analysis using β-actin as a loading control

Doxycycline (Dox)-inducible EGFRviii was stably established in F3 cells to generate Dox-inducible F3.EGFRviii cells, as described previously [10]. As shown in Fig. 1, even without Dox treatment, F3.EGFRviii cells showed morphological changes compared with F3 cells (Fig. 1a), due to slight leakage of EGFRviii expression in the absence of Dox (Additional file 2: Fig. S1). However, the morphology, typical of transformed cells (e.g., condensed cytoplasm, focus formation, and loss of contact inhibition) was clearly observed in F3.EGFRviii cells in response to Dox treatment, as previously reported (Fig. 1a, red line) [10]. That these alterations in cellular morphology were only observed after constant Dox treatment indicates that the low level of EGFRviii leakage in the absence of Dox (Additional file 2: Fig. S1) was insufficient to induce oncogenic transformation of F3.EGFRviii cells.

As expected, Dox treatment dramatically induced EGFRviii mRNA (Fig. 1b) and protein (Fig. 1c). When EGFRviii expression was induced by Dox treatment, active phosphorylation of EGFR (pY1068) was observed at the plasma membrane (white arrows, Fig. 1d). Therefore, we further examined the two crucial downstream pathways of EGFRviii, the PI3K/Akt and MEK/ERK1/2 pathways, by measuring the levels of phosphorylated Akt and ERK1/2, respectively. Akt was activated regardless of Dox treatment, which might result from leakage of EGFRviii expression (Additional file 2: Fig. S1). MEK/ERK1/2 activation became apparent after Dox treatment, along with EGFRviii expression (Fig. 1e).

### F3.EGFRviii as cancerous neural stem cells

**Fig. 2:**
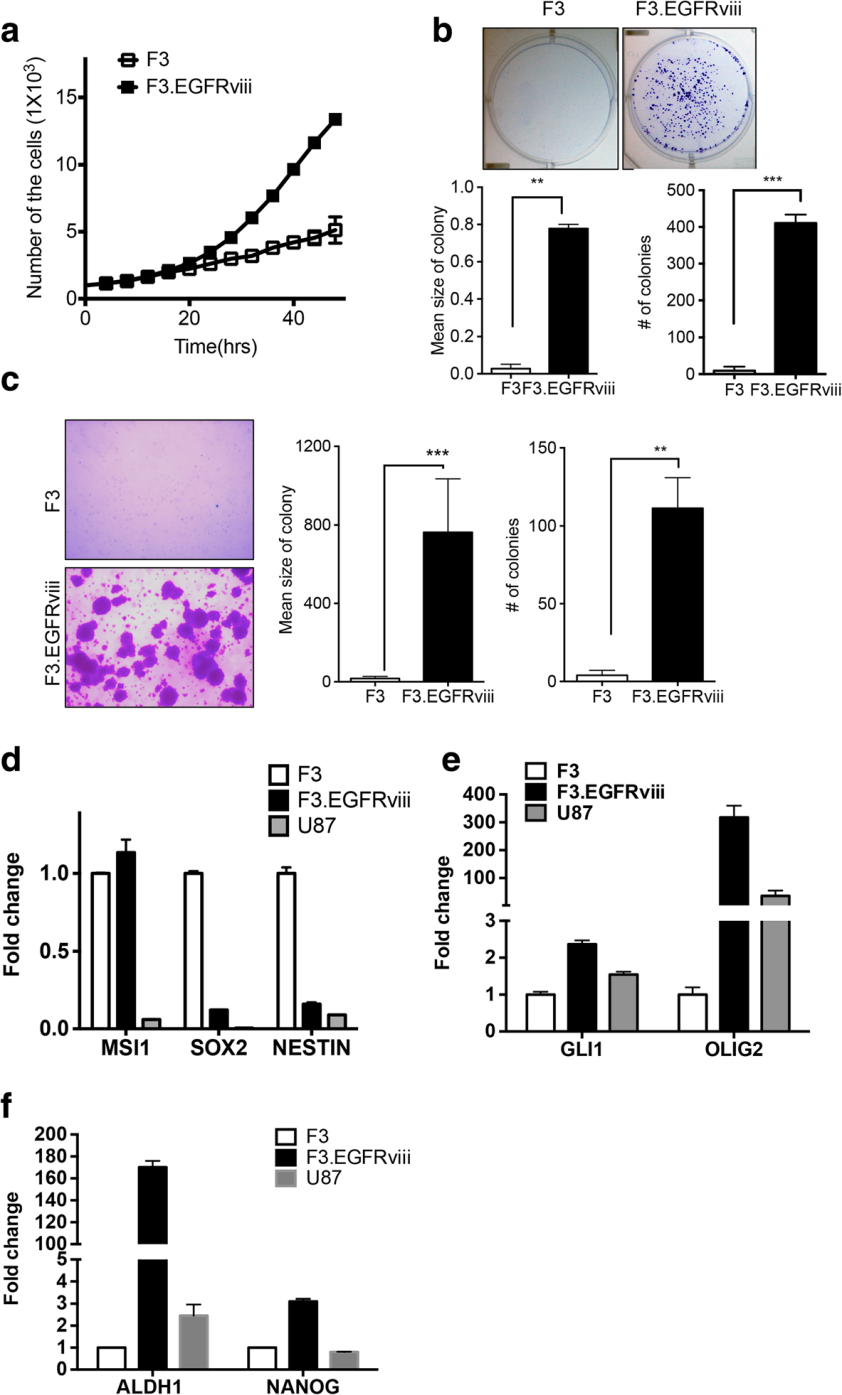
F3.EGFRviii cells as cancerous neural stem cells. **a** Accelerated proliferation of F3.EGFRviii cells relative to F3 cells was determined by cell counting at the indicated times. **b** Representative images from a clonogenic assay with F3 and F3.EGFRviii cells (*top panel*). The *graphs* show the mean number and size of colonies. **c** Images of colony formation in soft agar (*left panel*), and the colony number and mean colony size were presented (*right panel*). **d**-**f** The expression of neural stem cell and brain tumor markers in F3, F3.EGFRviii, and U87 cells was determined by real-time PCR (*n* = 3)

Due to the active phosphorylation of EGFRviii, followed by Akt and MEK/ERK1/2 activation (Fig. 1e), transformed F3.EGFRviii cells proliferated actively following repetitive Dox treatment (Fig. 2a) and formed clonogenic colonies from single cells (Fig. 2b). To test in vitro transformation capacity, anchorage-independent growth was determined using the soft agar assay. Consistently, the number of F3.EGFRviii colonies grown on soft agar was markedly increased compared with colonies arising from F3 cells, demonstrating that the F3.EGFRviii cells were transformed by repetitive Dox treatment (simply referred to as F3.EGFRviii cells from here on) (Fig. 2c). After undergoing oncogenic transformation, F3.EGFRviii cells retained the expression of musashi1 (*MSI1*) but not *SOX2* or *NESTIN*, none of which were highly expressed in U87 glioma cells (Fig. 2d). Instead, glioma-associated oncogene 1 (*GLI1*) and oligodendrocyte lineage transcription factor 2 (*OLIG2*), the expression of which is highly associated with glioma and GSCs [29], were upregulated (Fig. 2e). More importantly, nanog homeobox (*NANOG*) and aldehyde dehydrogenase 1 (*ALDH1*), which are both well characterized in GSCs [30, 31], were highly expressed in F3.EGFRviii cells (Fig. 2f).

To confirm that F3.EGFRviii cells retain neural stemness, CDr3, a commercially available NSC-specific florescent probe (NeuroFluor; StemCell Technologies), was used to stain F3 and F3.EGFRviii cells (Additional file 3: Fig. S2A). This probe effectively labeled both F3 and F3.EGFRviii cells, as expected. Alternatively, formation of secondary sphere, which is generally accepted to determine stemness (or self-renewal property) [32] was performed in F3.EGFRviii cells (Additional file 3: Fig. S2B). These results indicate that F3.EGFRviii cells retain neural cancer stemness, as in previous reports [6, 10].

### ERK1/2 activation in F3.EGFRviii spheres with increased cancer stemness

**Fig. 3:**
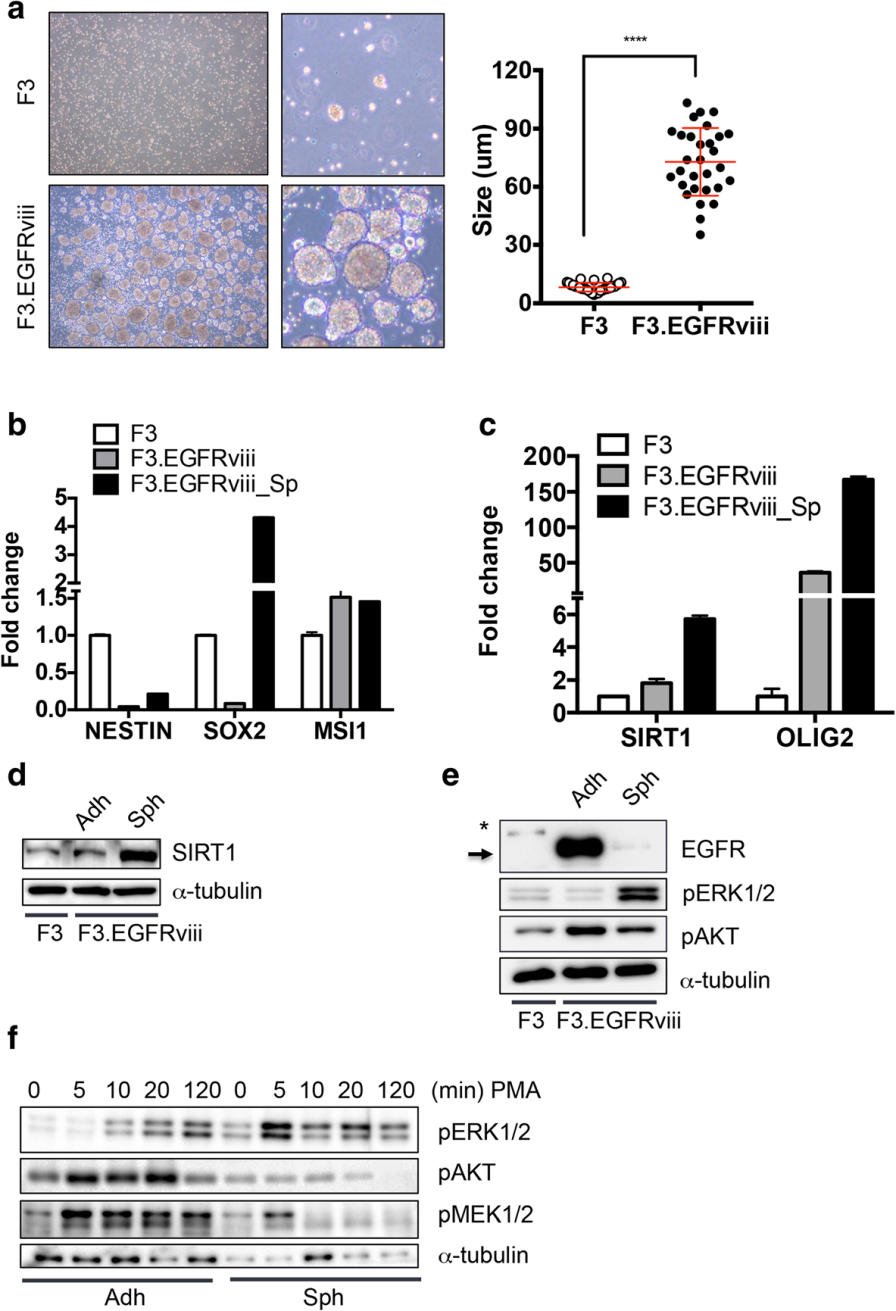
ERK1/2 activation in F3.EGFRviii spheres with increased cancer stemness. **a** Low- and high-magnification images of sphere formation in F3 and F3.EGFRviii cells after 14 days (*left panels*), and the mean sphere diameters from each cell type (*n* = 30, *right panel*). **b** and **c** Expression of the indicated genes in F3 cells, adherent F3.EGFRviii cells (Adh), and F3.EGFRviii spheres (Sph) was determined by real-time PCR (*n* = 3). **d** SIRT1 expression was determined by immunoblot analysis in F3 cells, adherent F3.EGFRviii cells (Adh), and F3.EGFRviii spheres (Sph) using α-tubulin as a loading control. **e** Immunoblot analyses of the indicated proteins in F3 cells, adherent F3.EGFRviii cells (Adh), and F3.EGFRviii spheres (Sph), with α-tubulin as a loading control (*, wild-type EGFR; *arrow*, expressed EGFRviii). **f** Adherent and spheroid F3.EGFRviii cells were treated with PMA (l μg/ml) and harvested at the indicated times for immunoblot analysis

The F3.EGFRviii cells were next maintained as sphere cultures to promote (or enrich) their neural cancer stemness. Interestingly, under sphere culture conditions, sphere formation was noticeably increased in F3.EGFRviii cells compared with control F3 cells (Fig. 3a), implying that cancer stemness was increased (or enriched) in F3.EGFRviii cells during sphere culture. To test this possibility, several neural cancer stemness markers were evaluated in F3, F3EGFRviii ahderent and F3.EGFRviii spheres. While *MSI1* expression was unaltered, *SOX2* was clearly induced in F3.EGFRviii cells under sphere culture conditions (Fig. 3b). Previously, we demonstrated that co-expression of *SOX2* and *SIRT1* is closely associated with neural cancer stemness [6]. Consistently, *SIRT1* expression was clearly increased in F3.EGFRviii spheres (Fig. 3c and d), along with *OLIG2*, which has recently been characterized in glioma [33] and GSCs [34]. In addition, F3.EGFRviii spheres were stained with CDr3, and levels of staining were compared with those of the adherent F3.EGFRviii cells. As shown in Additional file 3: Fig. S2C, intensity of CDr3 staining in F3.EGFRviii spheres was clearly increased. Given that CDr3 staining results from the expression of fatty acid-binding protein 7 (FABP7) [35], which is highly expressed in NSCs [36] and stem-like cells from GBMs [37], *FABP7* expression was examined and found to be clearly upregulated in F3.EGFRviii spheres compared with adherent F3.EGFRviii cells (Additional file 3: Fig. S2D). *SIRT1*, *SOX2*, and *OLIG2* were also upregulated in F3.EGFRviii spheres (Additional file 3: Fig. S2E). To further demonstrate the increased cancer stemness of F3.EGFRviii spheres, expression levels of typical cancer stemness genes were determined using a commercially available PCR array. Several genes, including *LIN28A* [38], *CD44* [39], *SOX2* [40], *CXCL8* (IL-8) [41], *MYCN* [42], *ID1* [43], and *KLF4* [44], all of which were previously reported to be overexpressed in GSCs, were significantly upregulated in F3.EGFRviii spheres compared with the adherent control cells (Additional file 3: Fig. S2F and G).

The adherent F3.EGFRviii cells showed high Akt activity but low ERK1/2 activity (Fig. 3e), while ERK1/2 activation was markedly increased in the F3.EGFRviii spheres, although EGFRviii induction by Dox was similar in adherent and spheroid cultures (Additional file 4: Fig. S3A). Of interest, we noticed that the protein expression of EGFRviii was remarkably lower in F3.EGFRviii spheres (Fig. 3e and Additional file 4: Fig. S3B) despite similar mRNA expression (Additional file 4: Fig. S3A). Treatment of cycloheximide (CHX) to F3.EGFRviii cells manifested decreased EGFR protein levels (Additional file 4: Fig. S3C), whereas treatment with the proteasome inhibitor MG132 or the autophagy inhibitor chloroquine (CQ) increased EGFR protein levels (Additional file 4: Fig. S3D), suggesting that downregulation of EGFRviii in F3.EGFRviii spheres may result from proteasomal or lysosomal degradation.

F3.EGFRviii cells were next treated with phorbol 12-myristate 13 acetate (PMA), which can activate Akt and ERK1/2 simultaneously through protein kinase C (PKC) [45, 46]. Interestingly, ERK1/2 signaling, but not Akt signaling, was markedly increased after PMA treatment in F3.EGFRviii spheres, while both Akt and ERK1/2 were activated in adherent F3.EGFRviii cells (Fig. 3f). These results suggest that a preference toward ERK1/2 signaling downstream of EGFRviii might be involved in maintaining neural cancer stemness.

### High expression of LAMTOR3 in F3.EGFRviii spheres for ERK1/2 activation

**Fig. 4:**
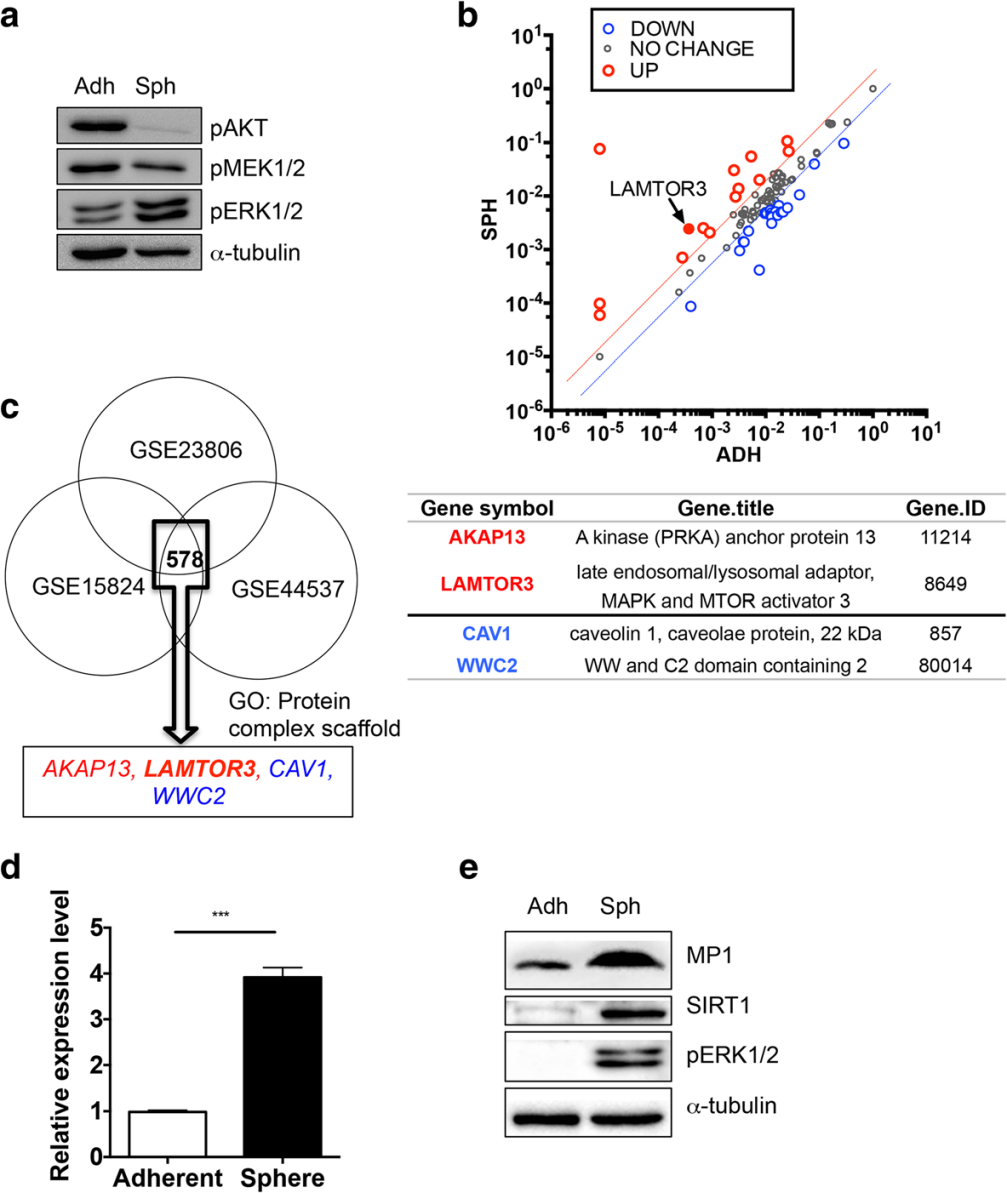
High expression of *LAMTOR3* in F3.EGFRviii spheres is associated with ERK1/2 activation. **a** The levels of pAKT, pMEK1/2, and pERK1/2 in adherent and spheroid F3.EGFRviii cells were determined by immunoblot analysis, using α-tubulin as a loading control. **b** Expression profile analysis of a MAPK signaling-associated gene set through MAPK pathway superarray in F3.EGFRviii cells and F3.EGFRviii spheres (*red*, genes upregulated >2-fold; *blue*, genes downregulated >2-fold; *gray*, no significant change). The *black arrow* indicates *LAMTOR3*. **c** GEO analysis to deduce candidate genes that distinguish signaling in F3.EGFRviii spheres. Three independent GSE studies, two using primary brain tumor cells (GSE23806, GSE15824) and one using induced cancer stem cells from neuroblastoma samples (GSE44537), were used to select commonly altered genes. Candidate genes were narrowed down using gene ontology for ‘protein complex scaffold’ genes. *Red* and *blue* indicate upregulated or downregulated genes, respectively. **d** The expression of *LAMTOR3* in adherent F3.EGFRviii cells and F3.EGFRviii spheres was determined by real-time PCR (*n* = 3). **e** The levels of MP1, SIRT1, and pERK1/2 were determined by immunoblotting in adherent and spheroid F3.EGFRviii cells using α-tubulin as a loading control

To examine the molecular mechanism underlying ERK1/2 activation in F3.EGFRviii spheres, the activity of MEK1/2, the sole upstream kinase for ERK1/2 [47], was examined. Surprisingly, the level of phosphorylation of MEK1/2 was similar between F3.EGFRviii spheres and adherent cells, although ERK1/2 activation was clearly enhanced in F3.EGFRviii spheres (Fig. 4a). We hypothesized that an increase in the expression of genes involved in the MEK/ERK1/2 signaling axis might be responsible for the increased ERK1/2 signaling in F3.EGFRviii spheres. To address this possibility, F3.EGFRviii cells were analyzed using a PCR array for the human MAPK signaling pathway to identify gene(s) involved in regulating the MEK/ERK1/2 signaling axis that was specifically altered in F3.EGFRviii spheres [48]. Among the 84 genes evaluated, 14 genes showed a significant change in expression in F3.EGFRviii spheres compared with adherent cells (Fig. 4b and Additional file 5: Fig. S4A). Next, to narrow down the possible candidates among those 14 genes, we searched the Gene Expression Omnibus database (http://www.ncbi.nlm.nih.gov/geo/) and chose three independent GSE studies, in primary brain cancer (GSE23806), primary glioblastoma (GSE15824), and neuroblastoma (GSE44537), that compared the gene expression signatures of glioma or GSCs with those of normal cells or glial tumors, respectively (Additional file 5: Fig. S4B). Among 578 genes with significantly altered expression [*p* < 0.05, 2-fold higher (shown in red) or lower (shown in blue)], putative gene candidates that may be responsible for ERK1/2 activation in F3.EGFRviii spheres were further refined based on the gene ontology term ‘protein complex scaffold.’ Four putative gene candidates [two downregulated genes (blue) and two upregulated genes (red)] were identified (Fig. 4c), and the relative expression levels of the four candidates in F3.EGFRviii spheres were confirmed in parallel with *SOX2*, which serves as a positive control for sphere formation (Additional file 5: Fig. S4C). Importantly, significant induction of *LAMTOR3*, the gene that encodes MEK partner-1 (MP1), which activates ERK1 [25] and ERK1/2 [27], was upregulated in F3.EGFRviii spheres in our PCR array (Fig. 4b), and was also upregulated in GSCs (or spheres) (Fig. 4c). In an independent experiment, the upregulation of *LAMTOR3* mRNA (Fig. 4d) and MP1 protein was confirmed in F3.EGFRviii spheres in parallel with high ERK1/2 phosphorylation and SIRT1 expression (Fig. 4e). Intriguingly, a positive correlation between *LAMTOR3* and *SIRT1* was also confirmed in a clinicogenomics database of GBM (http://betastasis.com/glioma/rembrandt) (Additional file 5: Fig. S4D). Additionally, analysis of a dataset of gene expression in GSCs compared with normal glioma cell lines (GSE23806) revealed that *LAMTOR3* expression was significantly higher in glioma cells with stemness (i.e., both glioma cells established by neurosphere culture and GSCs) in parallel with high *FABP7* and *SOX2* expression than in normal glioma cell lines (Additional file 5: Fig. S4E).

### LAMTOR3 expression and ERK activation in F3.EGFRviii cells

**Fig. 5:**
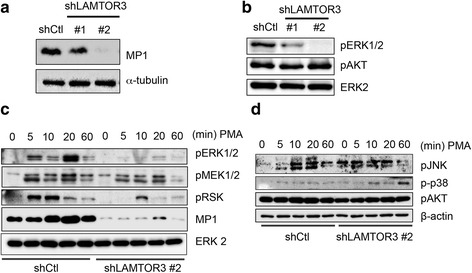
*LAMTOR3* expression and ERK activation in F3.EGFRviii cells. **a** MP1 expression was determined by immunoblot analysis of F3.EGFRviii cells transduced with a shRNA control lentivirus (shCtl) or a lentivirus expressing shRNA for *LAMTOR3* (sh*LAMTOR3* #1 and #2), using α-tubulin as a loading control. **b** The levels of pERK1/2 and pAKT were determined by immunoblotting in F3.EGFRviii cells. Total ERK2 was used as a loading control. **c** and **d** F3.EGFRviii cells were treated with PMA (l μg/ml) and harvested at the indicated times for immunoblot analysis using β-actin as a loading control

Considering the high expression of MP1 in F3.EGFRviii spheres, which showed high ERK1/2 activity, we hypothesized that MP1 expression may contribute to the ERK1/2 activation. To test this hypothesis, ERK1/2 activity was evaluated after depletion of MP1 from F3.EGFRviii cells. Among two different shRNA sequences for *LAMTOR3*, shLAMTOR#2 resulted in the clearest downregulation of the MP1 protein level (Fig. 5a and Additional file 6: Fig. S5A). As predicted, loss of MP1 expression was sufficient to significantly decrease the level of ERK1/2 phosphorylation in F3.EGFRviii cells (Fig. 5b). Importantly, the loss of MP1 resulted in only marginal effects on clonogenic growth (Additional file 6: Fig. S5B), anchorage-independent growth potential (Additional file 6: Fig. S5C), and the expression of *GLI1*, *OLIG2*, and *SIRT1* when F3.EGFRviii cells were grown in adherent cultures (Additional file 6: Fig. S5D). Next, cells were treated with PMA to stimulate Akt and MEK/ERK1/2 simultaneously, similar to the experiments shown in Fig. 3f. Interestingly, while MEK1/2 signaling remained intact after PMA treatment, activation of ERK1/2 and its downstream target S6 kinase (RSK) was clearly diminished (Fig. 5c). Activation of other MAPKs, such as p38 and JNK, and Akt signaling was only marginally affected by the loss of MP1 expression (Fig. 5d). These data suggest that MP1 expression may be critical for the shift in signaling preference toward ERK1/2 during F3.EGFRviii sphere formation, as shown in Fig. 3f.

### Role of LAMTOR3 in F3.EGFRviii spheres

**Fig. 6:**
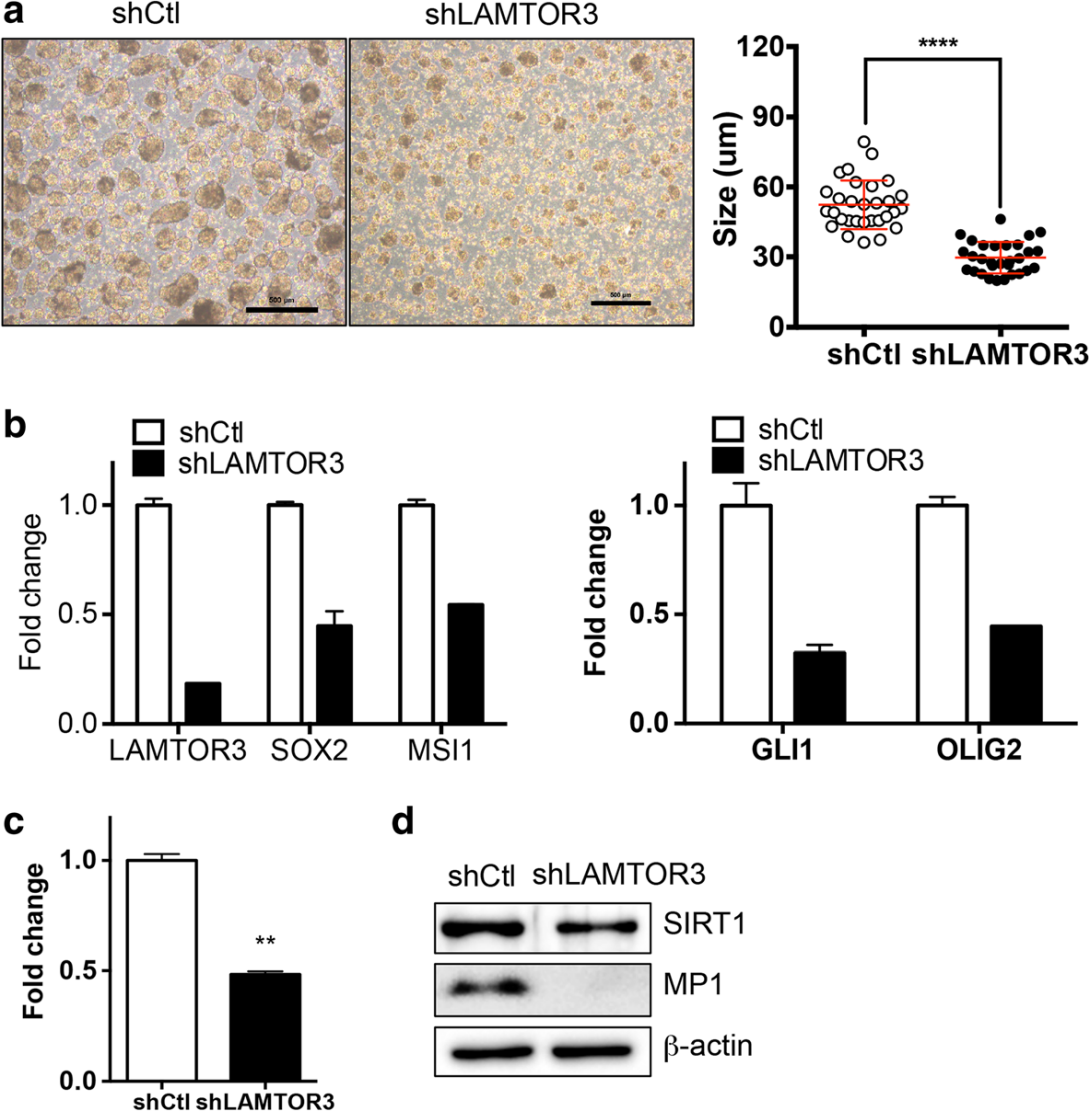
Role of *LAMTOR3* in F3.EGFRviii spheres. **a** Sphere formation with or without *LAMTOR3* expression was compared after 14 days (*left panel*). The graph shows the mean diameter of control and sh*LAMTOR3* spheres (*n* = 30, right panel). **b** and **c** The expression of the indicated genes was determined by real-time PCR (*n* = 2). **d** The levels of SIRT1 and MP1 were determined by immunoblotting using β-actin as a loading control

Considering the clear ERK1/2 activation during sphere formation in F3.EGFRviii cells and the role of MP1 in ERK activation in those cells, we speculated that the decrease in ERK1/2 activation caused by the depletion of MP1 might impair their sphere formation. To test this hypothesis, the sphere-forming capacity of F3.EGFRviii cells was evaluated after depletion of MP1. As predicted, *LAMTOR3* knockdown by either siRNA (Additional file 6: Fig. S5A) or shRNA (Fig. 6a) resulted in significantly impaired sphere formation of F3.EGFRviii cells. In parallel with the reduced sphere-forming capacity of F3.EGFRviii cells, self-renewality determined by formation of secondary sphere was also markedly reduced after loss of *LAMTOR3* (Additional file 7: Fig. S6B). Furthermore glial tumor markers such as *OLIG2* and *GLI1* and GSC markers such as *MSI1* and *SOX2* were also significantly reduced in F3.EGFRviii spheres (Fig. 6b). SIRT1, the expression of which is important for neural stemness as well as neural cancer stemness and survival [6], was also markedly reduced at both the mRNA (Fig. 6c) and protein (Fig. 6d) level by depletion of MP1.

### Prognostic significance of LAMTOR3 in brain tumors

**Fig. 7:**
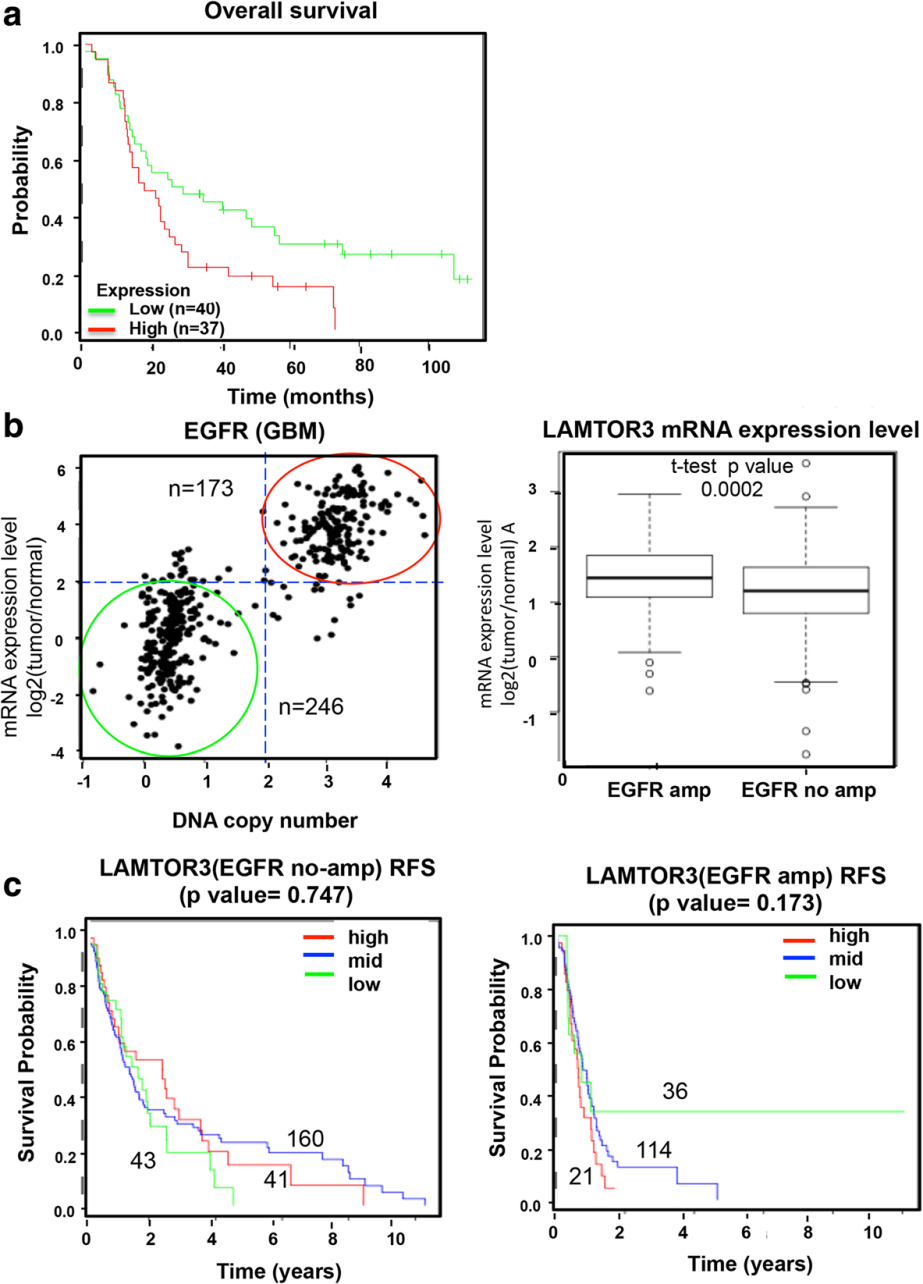
Prognostic significance of *LAMTOR3* expression in brain tumors. **a** A Kaplan-Meier survival analysis was performed according to *LAMTOR3* expression. **b** and **c** Data from the TCGA database were analyzed. **b** GBM samples (*n* = 419) were separated into two groups based on DNA copy number and mRNA expression of *EGFR* (*left panel*). The samples indicated by the red line were considered to have EGFR amplification (*n* = 173), while the samples indicated by the green line were considered to have no EGFR amplification (*n* = 246) (*left panel*). The expression of *LAMTOR3* in the two groups is shown in the right panel (*p* < 0.0002). **c** Kaplan-Meier relapse-free survival analyses according to *LAMTOR3* in the patients without (*left panel*) or with (*right panel*) EGFR amplification GBM samples (*right panel*)

To investigate the prognostic significance of MP1, a survival analysis was performed in high-grade glioma with data from a publicly available clinicogenomics study (GSE4271) [49] using DRUGSURV (http://www.bioprofiling.de/cgi-bin/GEO/DRUGSURV/start_GENE.pl). As shown in Fig. 7a, high *LAMTOR3* expression was largely correlated with poor clinical outcome in 77 patients (*p* = 0.122). The clinical significance of *LAMTOR3* in relation to EGFR amplification was further investigated using a large GBM dataset from The Cancer Genome Atlas (TCGA). The glioblastoma samples were categorized into two subgroups based on the DNA copy number and mRNA expression level of EGFR. Among 500 glioblastoma samples in TCGA, 173 samples were categorized as EGFR-amplified samples (red circle), and 246 samples (green circle) with normal or low DNA copy numbers and EGFR expression were categorized as EGFR-normal samples (Fig. 7a). *LAMTOR3* expression was significantly higher in the EGFR-amplified group (*p* = 0.0002) (Fig. 7b). The effects of *LAMTOR3* expression on survival also differed according to EGFR status. In the EGFR-amplified group, the probability of survival was significantly better in samples with low *LAMTOR3* expression, but there were no significant differences in survival according to *LAMTOR3* expression among the samples without EGFR amplification (Fig. 7c).

## Discussion

Previously, we established CNSCs from F3 cells using the H-RasV12 oncogene [10], and demonstrated that SIRT1 expression is important for maintaining neural cancer stemness in CNSCs and GSCs [6]. Considering the frequent gain-of-function mutations of EGFR in glioma, characterization of CNSCs expressing EGFRviii is important for understanding the molecular properties of neural cancer stemness.

F3.EGFRviii cells showed the typical transformed cell phenotypes, such as clear morphological changes (Fig. 1a), accelerated growth (Fig. 2a), and anchorage-independent growth (Fig. 2c), and readily formed spheres with high induction of neural cancer stemness markers including *SOX2*, *SIRT1*, and *OLIG2*. Interestingly, while activation of Akt rather than MEK/ERK1/2 following EGFRviii expression appeared to be dominant in adherent cells (Fig. 1e), ERK1/2 was highly activated when F3.EGFRviii cells were subjected to sphere formation (Fig. 3e), suggesting that neural cancer stemness is associated with a switch in preference from Akt signaling to ERK1/2 signaling after EGFR activation. Induction of *LAMTOR3* was found in GSCs or GSLCs, which have been described as cells with neural cancer stemness (Fig. 4c and Additional file 5: Fig. S4E), and was also identified as an important mediator for ERK1/2 activation following EGFR stimulation (Fig. 5). Thus, depletion of *LAMTOR3* in F3.EGFRviii cells decoupled EGFR signaling from ERK1/2, thereby impairing sphere formation and attenuating the expression of genes associated with neural cancer stemness (Fig. 6), despite only marginal effects on signaling to MEK1/2 and other MAPKs (Fig. 5c and d).

Considering that the PI3K/Akt and MEK/ERK1/2 pathways are two major downstream signaling pathways of activated EGFR in glioma [50], therapeutic approaches for the inhibition of these signaling pathways are being actively studied [24]. Indeed, ERK activity has been found to be highly elevated in glioma under EGFR amplification [51], while Akt activation is also frequent [52] and has been shown to be important for neural cancer stemness [53]. However, it is still unclear how ERK1/2 activity remained elevated despite clear downregulation of EGFRviii in F3.EGFRviii sphere (Fig. 3e). To resolve this discrepancy, MAP kinase signaling pathway PCR array was applied. Of interest, V-Mos Moloney Murine Sarcoma Viral Oncogene Homolog (MOS), which is responsible for ERK activation as a MAPK kinase kinase (MAPK3K) level in meiosis [54], was upregulated in F3.EGFRviii sphere (Additional file 5: Fig. S4A) as consistent as previous reports the high expression of MOS in ependymal glioma [55] and astrocytic tumors [56]. Alternatively, *LAMTOR3*, enabling ERK1 activation [25] as a scaffolding protein was highly upregulated in F3.EGFRviii sphere (Additional file 5: Fig. S4A). Notably, recent studies reveal that *LAMTOR3* expression mediates ERK1/2 activation independently of mutations in *RAS*, thereby contributing to pancreatic tumorigenesis [27]. This finding implies that *LAMTOR3* controls oncogenic signals in addition to mediating endosomal signaling [57]. In addition, recent studies show that ERK1/2 activity promotes chemo- and radio-resistance in GBM [51]. Consistently, poor prognosis in patients with high *LAMTOR3* was even more prominent after chemotherapy (http://betastasis.com/glioma/rembrandt, data not shown).

The finding that neural cancer stemness correlates with a clear signaling preference for either Akt or ERK following EGFRviii activation suggests that the dependency of GBM for either Akt or ERK may depend on the expression of *LAMTOR3*. TCGA analysis revealed that *LAMTOR3* expression in GBM patients was significantly higher when accompanied by gain-of-function mutations in EGFR, and was correlated with poor prognosis (Fig. 7). These findings suggest that high *LAMTOR3* expression in GBM may promote EGFR downstream signaling through ERK.

Collectively, these studies used a model of EGFRviii-expressing CNSCs to reveal novel insights into the molecular mechanisms involved in maintaining neural cancer stemness.

## Conclusions

Using F3.EGFRviii CNSCs model, we demonstrated *LAMTOR3* in GBM served as an important signaling mediator to control MEK1/ERK1/2 pathway, of which activation contributed to maintaining ‘neural cancer stemness’.

## Additional files

Additional file 1: Table S1. Oligonucleotide primer sequences. (TIFF 175 kb)

Additional file 2: EGFRviii expression in Dox inducible F3.EGFRviii cell line. (TIFF 74 kb)

Additional file 3: Augmented cancer stemness in F3.EGFRviii sphere. (ZIP 2262 kb)

Additional file 4: Protein stability of EGFR in F3.EGFRviii sphere. (TIFF 423 kb)

Additional file 5: High expression of LAMTOR3 in F3.EGFRviii sphere. (ZIP 525 kb)

Additional file 6: Role of LAMTOR3 in cancerous neural stem cell maintenance. (TIFF 1742 kb)

Additional file 7: Role of LAMTOR3 in F3.EGFRviii sphere formation. (TIFF 2923 kb)

## Abbreviations

EGFR: Epidermal growth factor receptor
ERK: Extracellular signal–regulated kinase
GBM: Glioblastoma multiform
MEK: Mitogen-activated protein Kinase kinase 1 or 2
MP1: MEK partner-1
RFS: Recurrence free survival
