# Supplementary figures and images for: Role of MEK partner-1 in cancer stemness through MEK/ERK pathway in cancerous neural stem cells, expressing EGFRviii
Source: Mol Cancer. 2017 Aug 22;16:140. doi: 10.1186/s12943-017-0703-y (PMC5567886; doi:10.1186/s12943-017-0703-y)

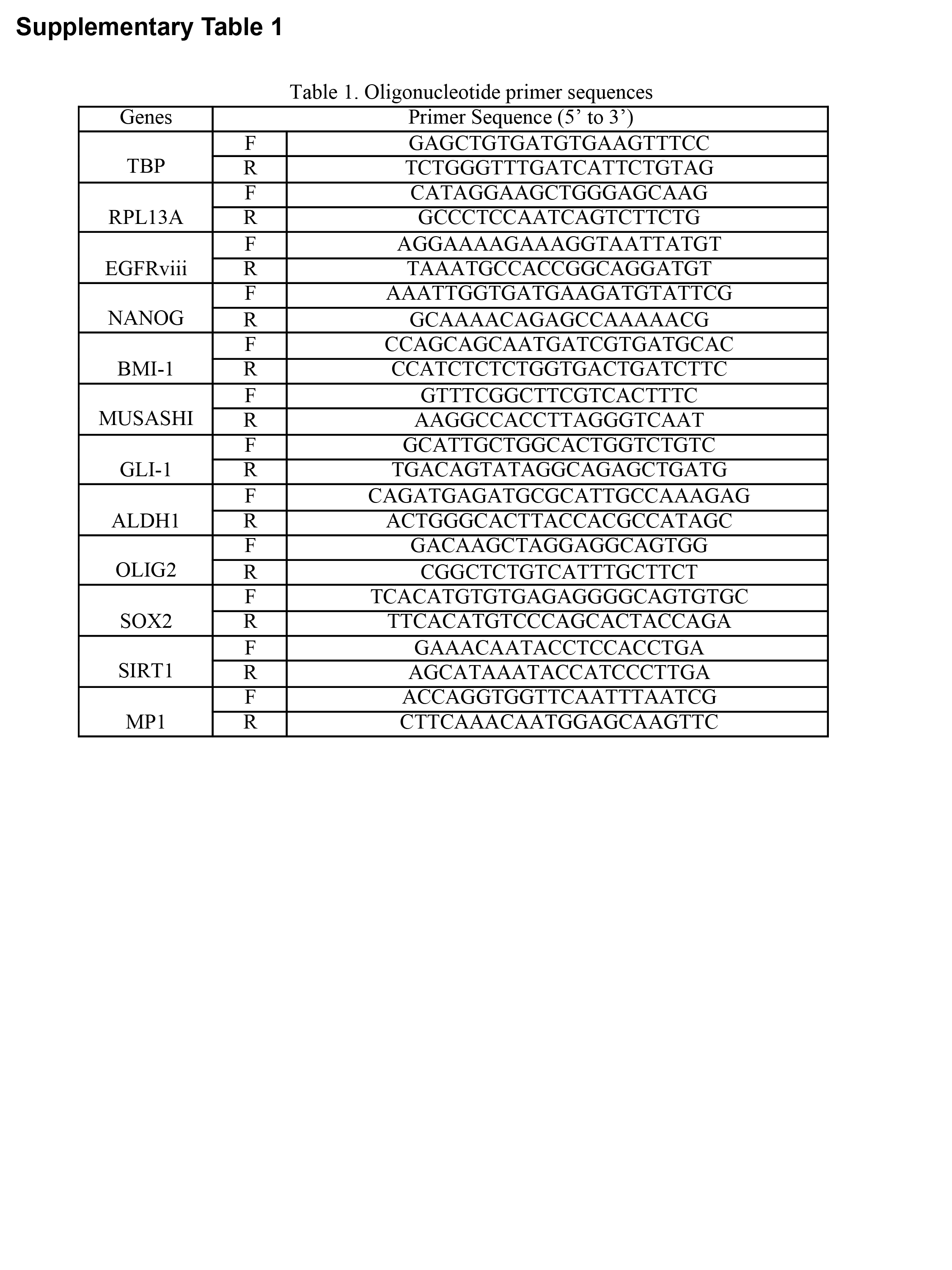

Supplement: Supplementary file 1 — Oligonucleotide primer sequences. (TIFF 175 kb) [file 12943_2017_703_MOESM1_ESM.tif]

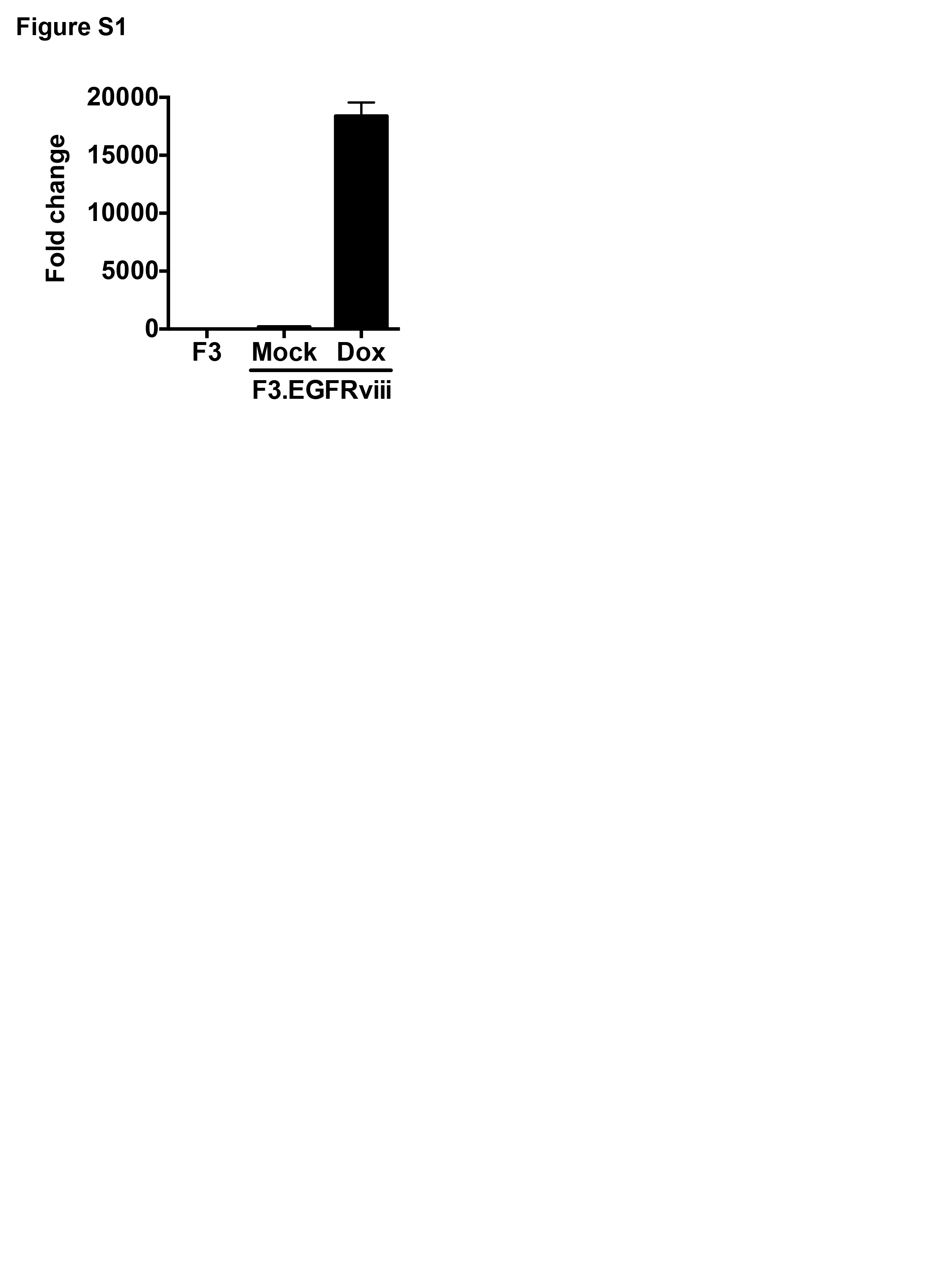

Supplement: Supplementary file 2 — EGFRviii expression in Dox inducible F3.EGFRviii cell line. (TIFF 74 kb) [file 12943_2017_703_MOESM2_ESM.tif]

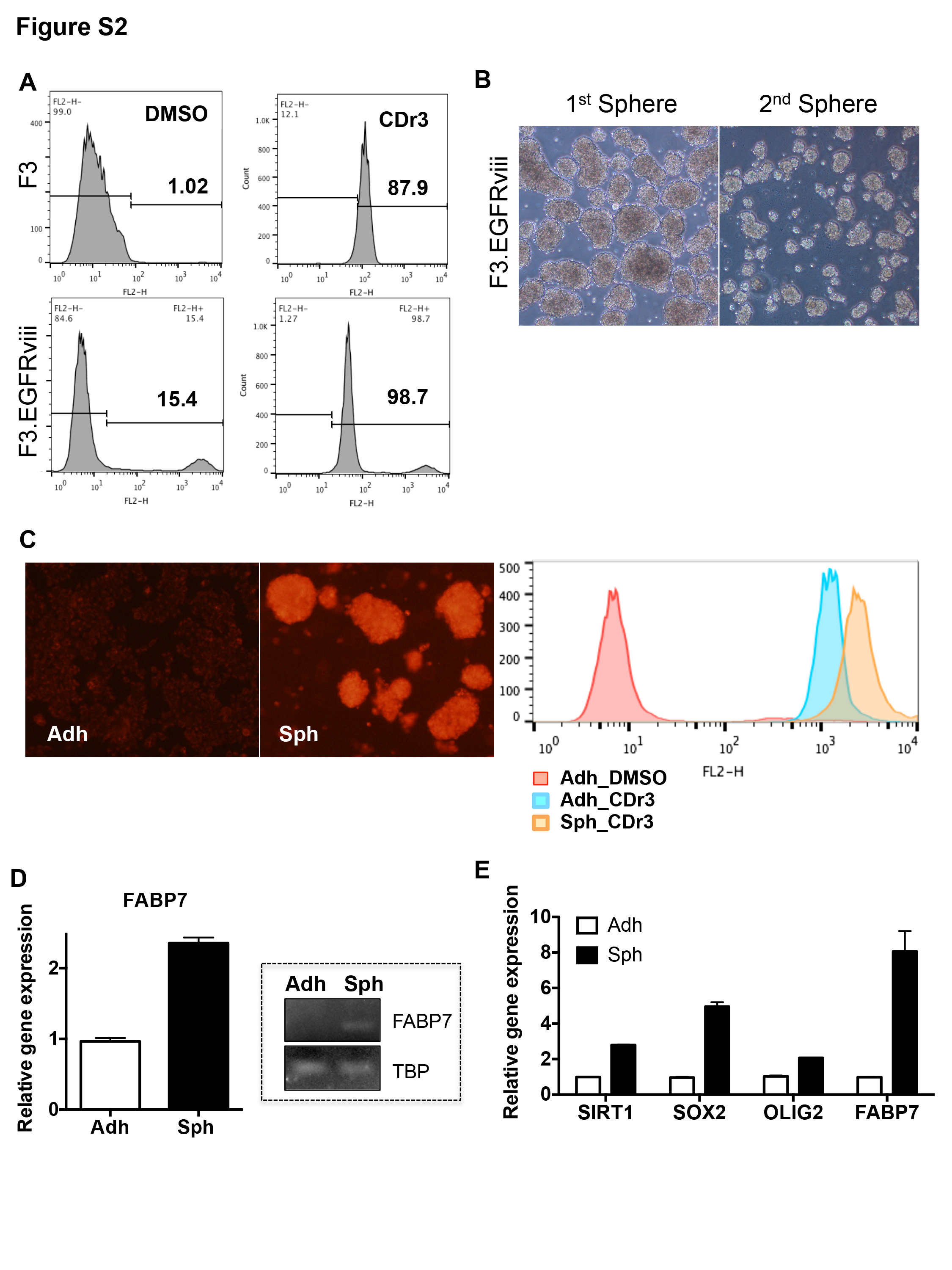

Supplement: Supplementary file 3 — Augmented cancer stemness in F3.EGFRviii sphere. (ZIP 2262 kb) [file 12943_2017_703_MOESM3_ESM.zip › Fig S2.tif]

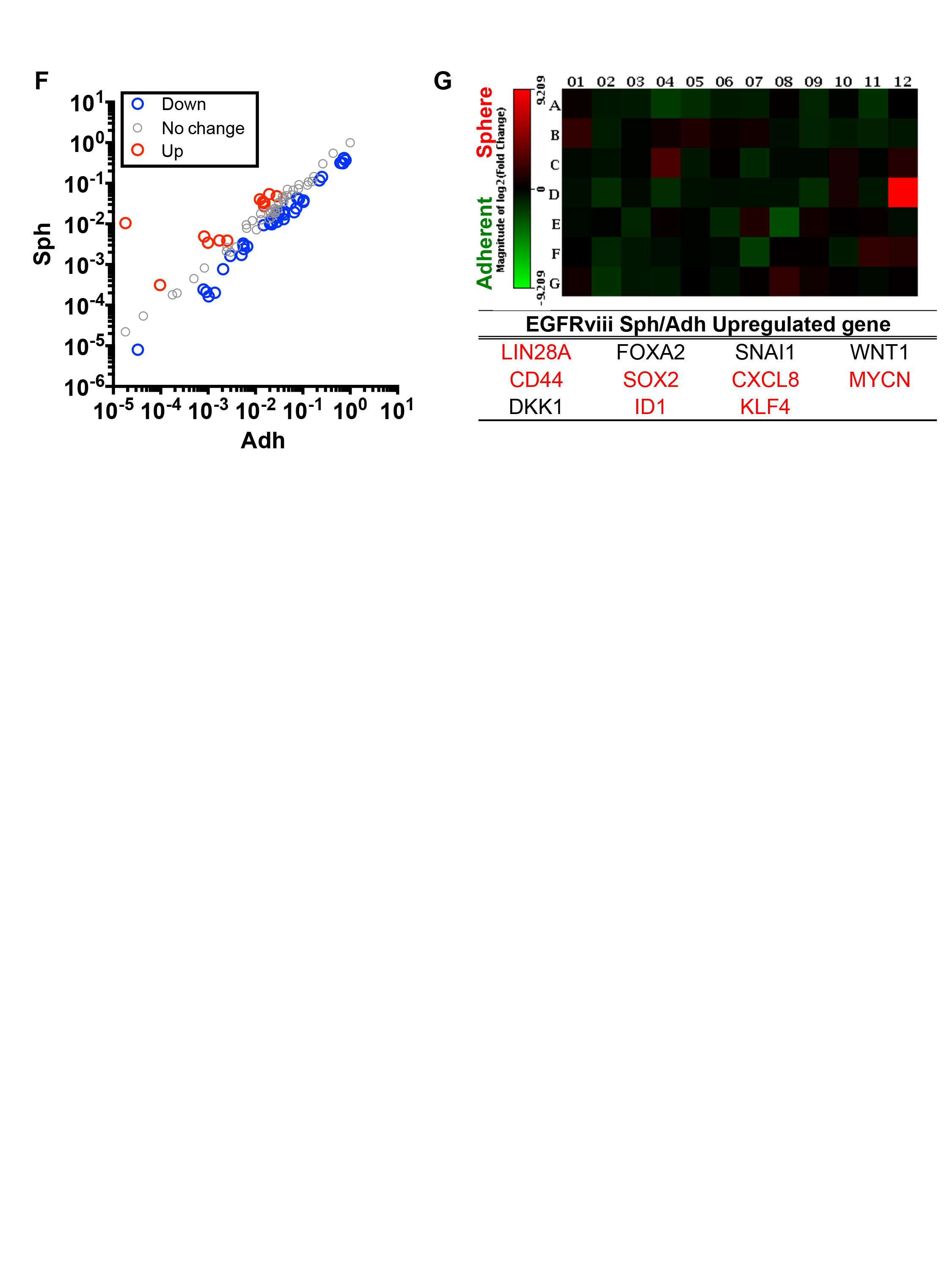

Supplement: Supplementary file 3 — Augmented cancer stemness in F3.EGFRviii sphere. (ZIP 2262 kb) [file 12943_2017_703_MOESM3_ESM.zip › Fig S2FG.tif]

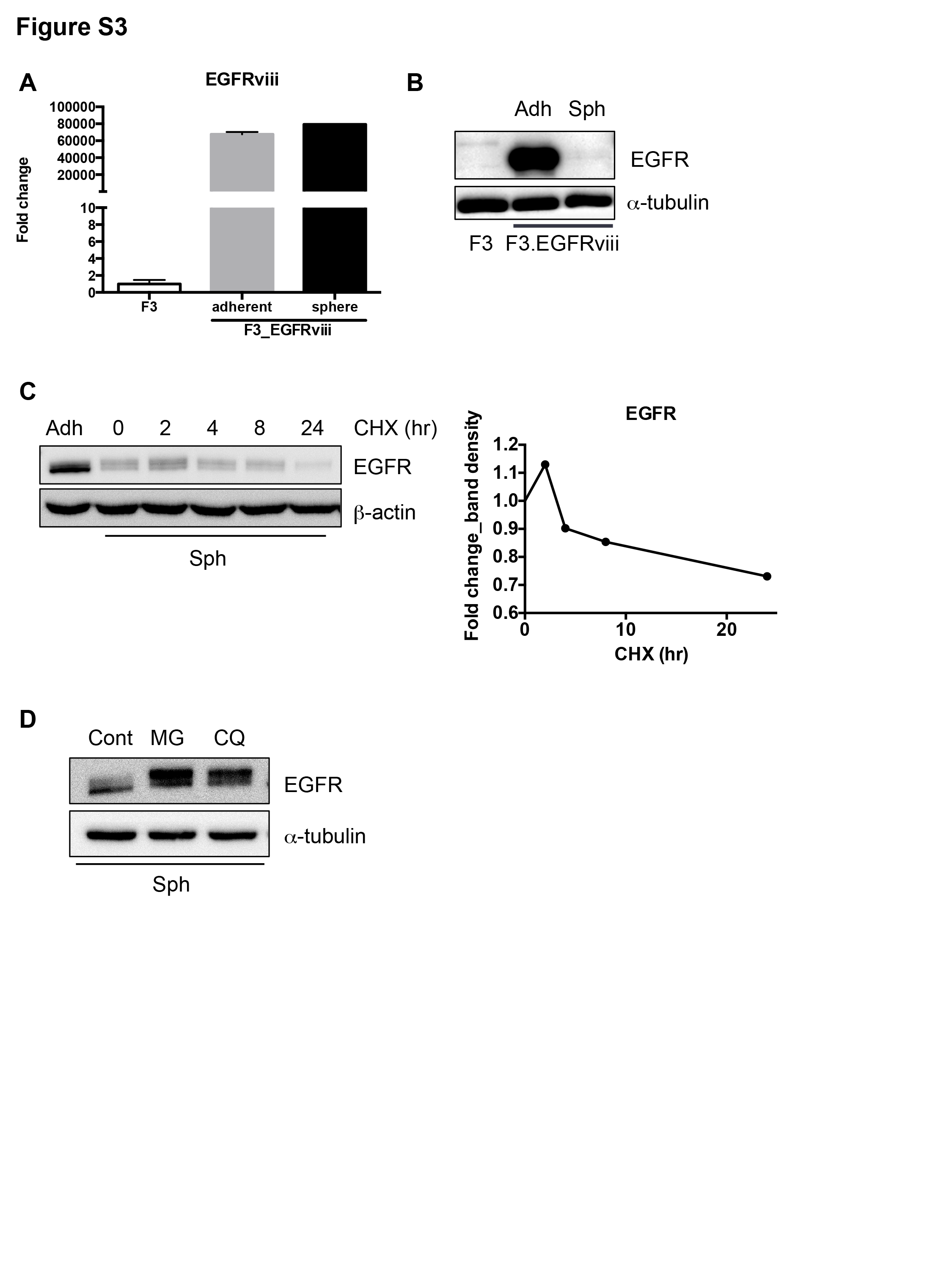

Supplement: Supplementary file 4 — Protein stability of EGFR in F3.EGFRviii sphere. (TIFF 423 kb) [file 12943_2017_703_MOESM4_ESM.tif]

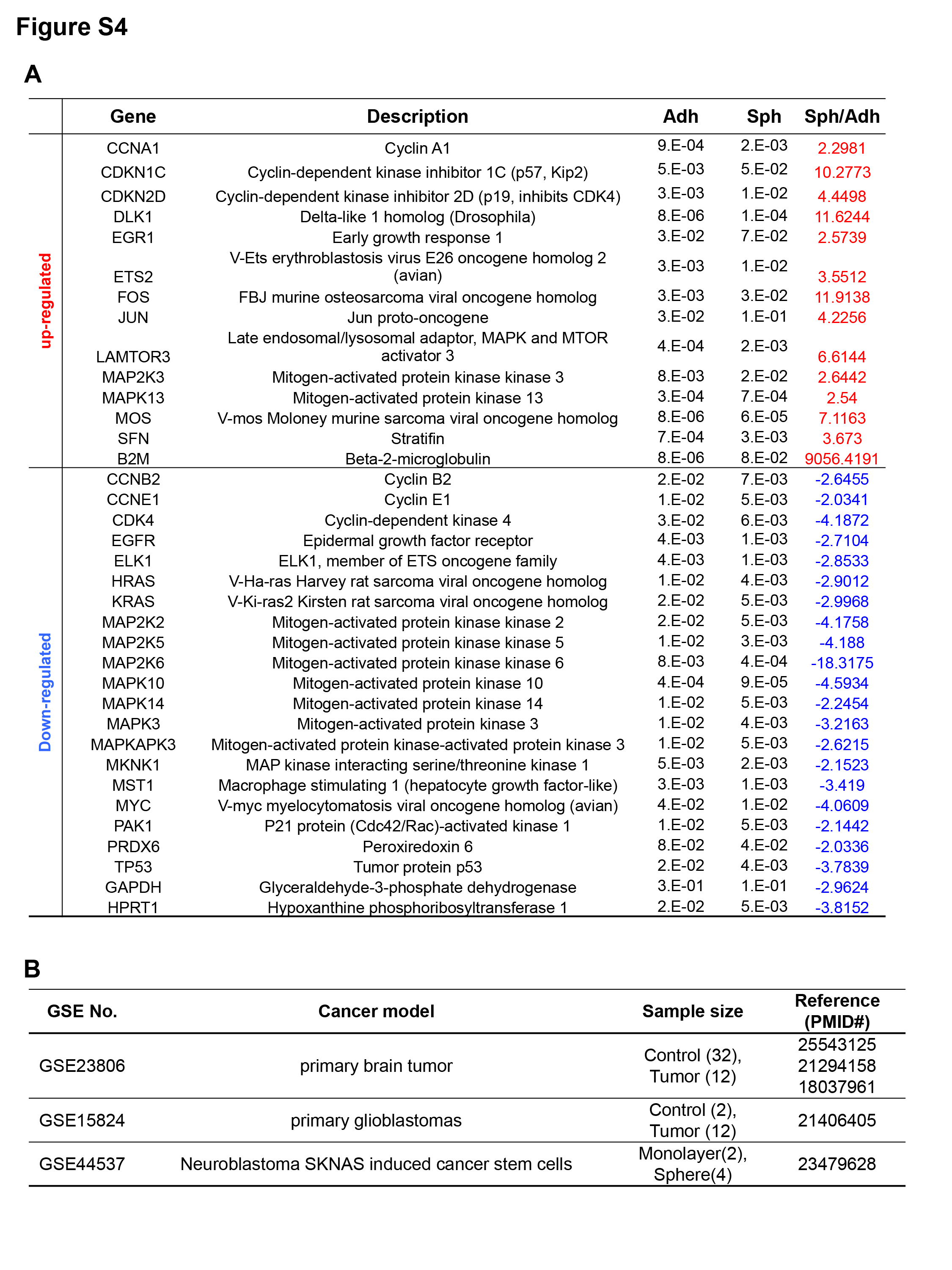

Supplement: Supplementary file 5 — High expression of LAMTOR3 in F3.EGFRviii sphere. (ZIP 525 kb) [file 12943_2017_703_MOESM5_ESM.zip › Fig S4.tif]

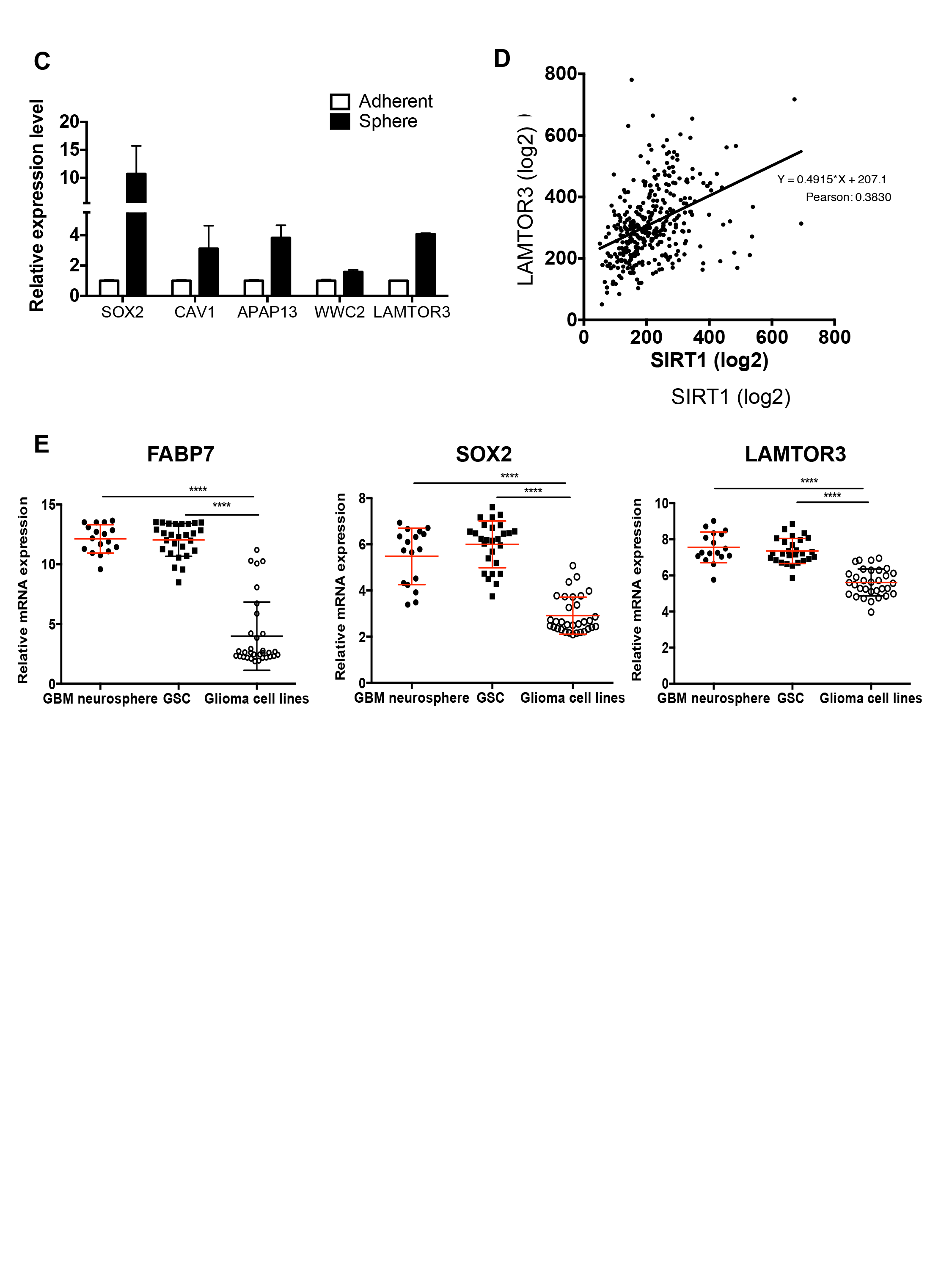

Supplement: Supplementary file 5 — High expression of LAMTOR3 in F3.EGFRviii sphere. (ZIP 525 kb) [file 12943_2017_703_MOESM5_ESM.zip › Fig S4CDE.tif]

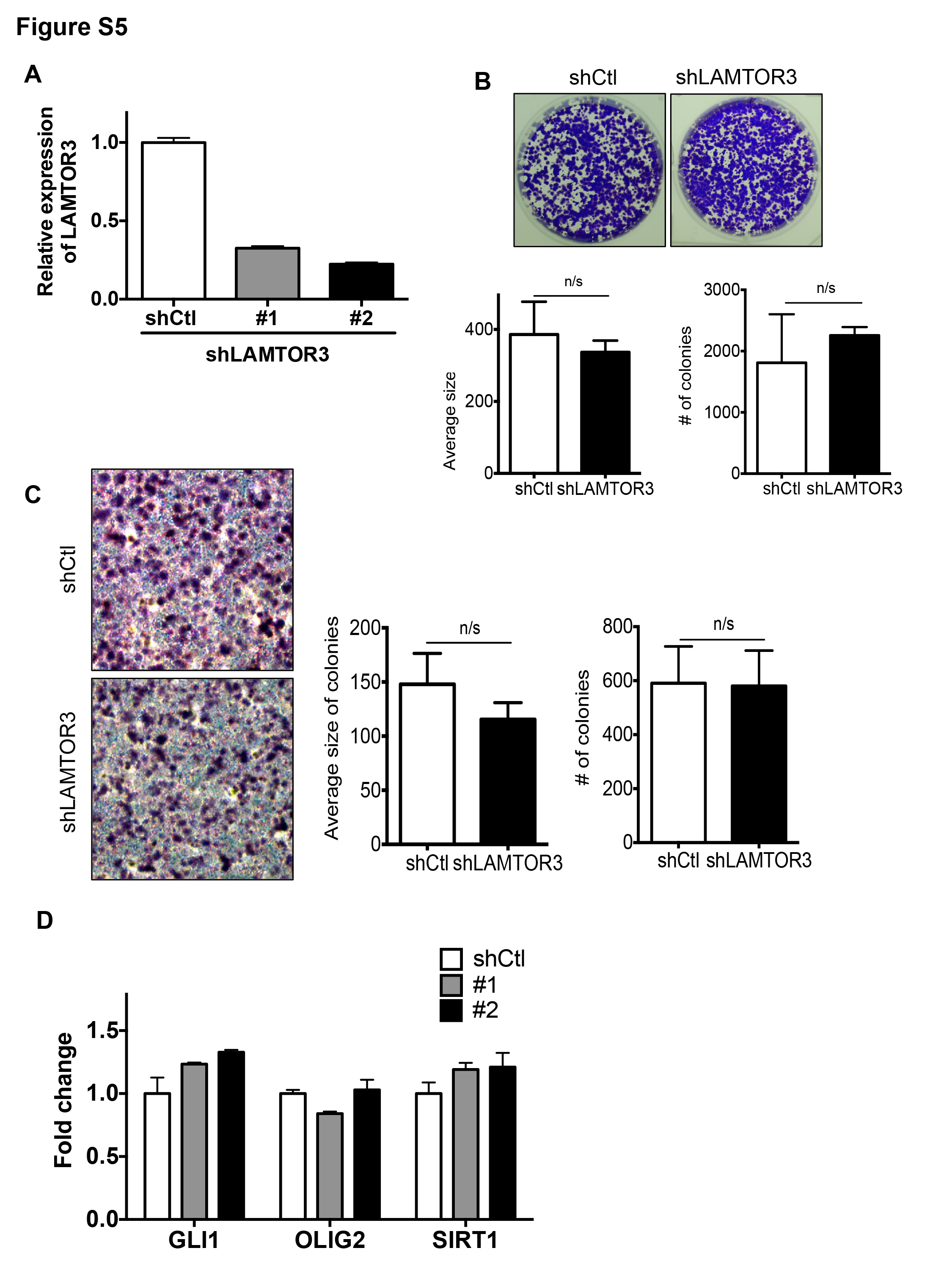

Supplement: Supplementary file 6 — Role of LAMTOR3 in cancerous neural stem cell maintenance. (TIFF 1742 kb) [file 12943_2017_703_MOESM6_ESM.tif]

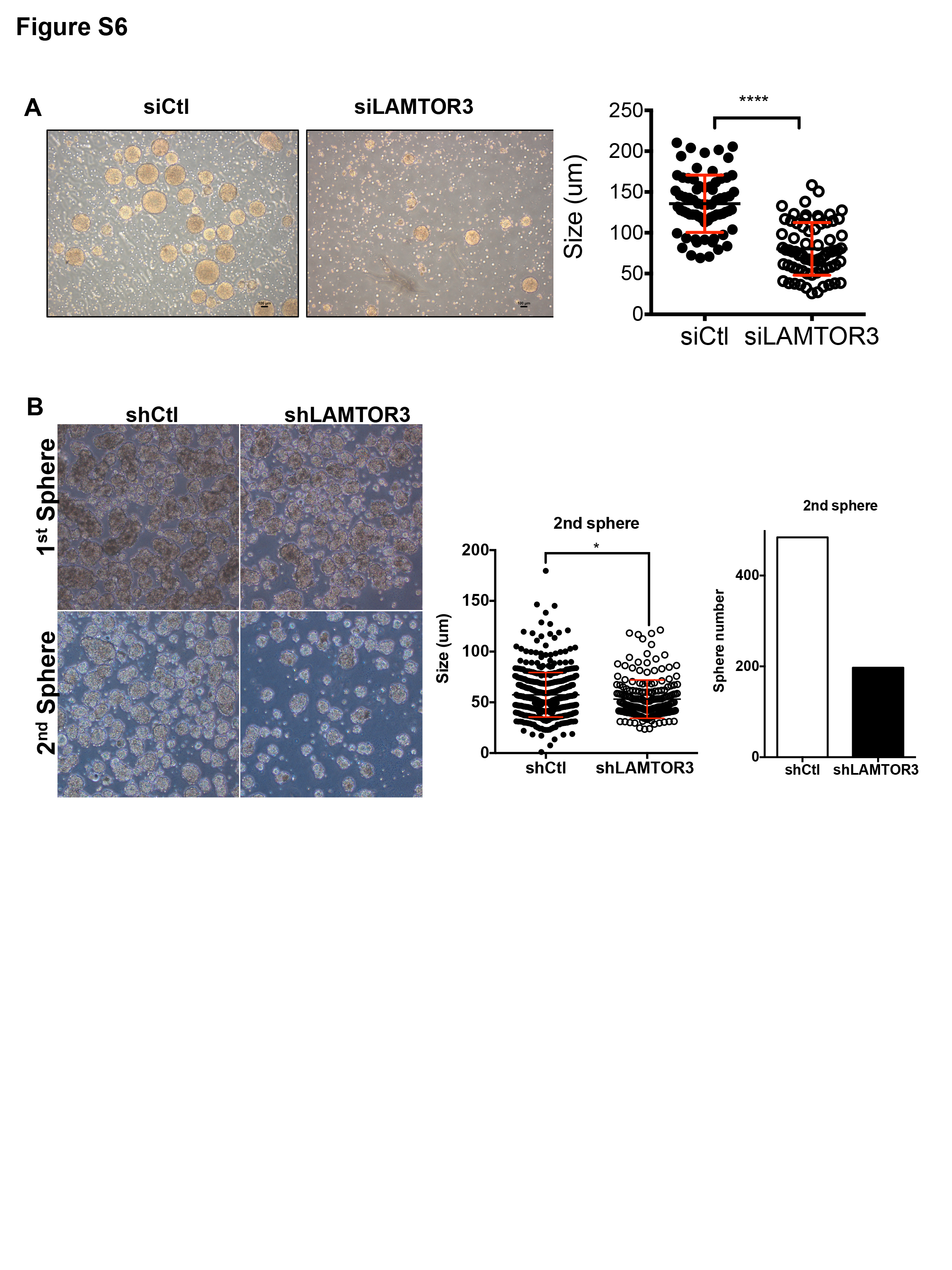

Supplement: Supplementary file 7 — Role of LAMTOR3 in F3.EGFRviii sphere formation. (TIFF 2923 kb) [file 12943_2017_703_MOESM7_ESM.tif]
